# Effective Extraction and Determination of 3-Nitropropanoic Acid in Sugarcane Samples Based on Magnetic Solid-Phase Extraction Coupled with HPLC-MS/MS

**DOI:** 10.3390/foods15193466

**Published:** 2026-09-28

**Authors:** Yaqi Zhang, Jing Dai, Yijing Zhao, Aibo Wu, Lijun He, Lei Zheng, Wei Min, Jiang Liang, Na Liu, Lipeng Hao

**Affiliations:** 1 Shanghai Pudong New Area Center for Disease Control and Prevention (Shanghai Pudong New Area Health Supervision Institute), Shanghai 200136, China; 2 Shanghai Institute of Nutrition and Health, University of Chinese Academy of Sciences, Chinese Academy of Sciences, Shanghai 200031, China; 3 School of Chemistry and Chemical Engineering, Henan University of Technology, Zhengzhou 450001, China; 4 China National Center for Food Safety Risk Assessment, Beijing 100022, China

**Keywords:** 3-nitropropanoic acid, sugarcane, magnetic solid-phase extraction, MCX, HPLC-MS/MS

## Abstract

To reduce the risk associated with 3-nitropropanoic acid (3-NPA) contamination, an efficient and accurate analytical method for 3-NPA determination in sugarcane samples was established by combining magnetic solid-phase extraction (MSPE) with high-performance liquid chromatography–tandem mass spectrometry (HPLC-MS/MS). Owing to the hydrogen bonding and hydrophobic interactions, the mixed strong cation exchange (MCX) magnetic adsorbent showed effective extraction ability toward 3-NPA. Only 2 mL of organic solvent was consumed during the MSPE procedure, and the operation time was short (5 min). The limits of detection were 0.02 μg kg^−1^ for sugarcane juice and 0.04 μg kg^−1^ for sugarcane. The developed method showed satisfactory spiked relative recoveries (96.9–105.4%) and good precision values (RSD 1.4–9.7%). MCX-MSPE cleanup provided partial mitigation of matrix suppression, with the absolute matrix effect value rising from 39.4% to 57.1%. This study provides an efficient analytical alternative for the determination of 3-NPA in sugarcane samples.

## 1. Introduction

3-Nitropropanoic acid (3-NPA) is a neurotoxin produced by *Arthrinium* and is frequently found in mouldy sugarcane, corn, and coconut, as well as related products [1,2,3]. As a naturally occurring neurotoxin, 3-NPA can cause human intoxication with symptoms varying from vomiting and abdominal pain to dystonia, coma, and even death [4,5]. With the intensification of global climate change, the occurrence of 3-NPA contamination events has increased noticeably worldwide. In Serbia, the contamination level of 3-NPA in corn reached up to 44% under hot and dry climatic conditions [2]. In China, 3-NPA present in mouldy sugarcane has already been recognized as an etiological factor causing intoxication [1,6]. As one of the major sugarcane-producing countries, China urgently requires effective monitoring of 3-NPA contamination to guarantee food safety and public health. However, no standardized analytical method has yet been established for 3-NPA determination, which seriously limits accurate and efficient monitoring of 3-NPA contamination in sugarcane.

In recent years, several analytical methods have been developed for 3-NPA detection, including fluorescence sensing, electrochemical techniques, gas chromatography–atmospheric pressure chemical ionization–tandem mass spectrometry (GC-APCI-MS/MS), and high-performance liquid chromatography–tandem mass spectrometry (HPLC-MS/MS) [1,2,7,8]. Although fluorescence sensing and electrochemical approaches demonstrated high sensitivity and fast response, their practical application is easily affected by interference from complex food matrices [9]. GC-MS/MS analysis requires derivatization of 3-NPA, a process that is laborious, time-consuming, and may introduce additional analytical variability [6]. In contrast, HPLC-MS/MS analysis does not require derivatization and can be performed directly [2,10,11]. Nevertheless, the accurate and reliable quantification of 3-NPA in complex food matrices remains a challenge. Due to its high polarity, low molecular weight and hydrophilic character, with a calculated octanol-water partition coefficient (XlogP) of −0.5, 3-NPA exhibits poor retention on reversed-phase chromatographic columns, which readily leads to co-elution with matrix components. This, combined with pronounced matrix effects (primarily ion suppression), low natural occurrence levels, and the absence of commercially available isotopic internal standards, makes it difficult to achieve accurate results through instrumental parameter optimization alone [6,12]. Accordingly, effective sample pretreatment and cleanup are critical for robust and accurate determination of 3-NPA.

Solid-phase extraction (SPE) is widely used as a pretreatment approach for 3-NPA determination. Primary secondary amine (PSA) and NH_2_ SPE columns used in earlier studies generally exhibited limited selectivity, resulting in matrix interference and decreased analytical accuracy [13,14]. Furthermore, ammonium hydroxide was needed during elution, but alkaline conditions could induce degradation of 3-NPA, thereby reducing method stability and reproducibility. In addition, conventional SPE procedures are time-consuming (30–60 min), labor-intensive, and dependent on specialized equipment, which greatly restricts their use in high-throughput analysis [15]. Recently, magnetic solid-phase extraction (MSPE) has received extensive interest because of its simple operation, rapid processing, high extraction efficiency, and low consumption of organic solvents [16,17]. To date, only one report has described the use of MSPE for 3-NPA pretreatment, in which molecularly imprinted polymers were used as selective adsorbents [7]. However, the synthesis of molecularly imprinted polymers is relatively complicated, making standardization and reproducibility among laboratories difficult [18]. In comparison, commercial magnetic adsorbents possess good stability and broad application potential, but their use in 3-NPA sample pretreatment has not yet been reported. Therefore, systematic studies are still needed to assess the extraction performance of commercial magnetic adsorbents for 3-NPA during MSPE and to clarify the extraction mechanism in complicated sugarcane matrices.

In this work, an efficient and accurate method for 3-NPA determination in sugarcane and sugarcane juice was first established by integrating functionalized magnetic adsorbent-based MSPE with HPLC-MS/MS. The extraction conditions and MSPE procedure were systematically optimized. Among the tested adsorbents, the mixed strong cation exchange (MCX) magnetic adsorbent exhibited the best extraction performance for 3-NPA together with strong resistance against matrix interference, representing the first report of its excellent applicability in 3-NPA determination. Moreover, the performance of the established method was evaluated through determination of 3-NPA in sugarcane and sugarcane juice samples. This study provides a new analytical strategy for efficient, sensitive, and accurate determination of 3-NPA.

## 2. Materials and Methods

### 2.1. Reagents and Materials

The 3-NPA standard was purchased from Pribolab Scientific Co., Ltd. (Qingdao, China). A stock solution (10 μg mL^−1^) was prepared using methanol and stored at −18 °C. Magnetic adsorbents, including MCX, mixed strong anion exchange (MAX), PSA, hydrophilic interaction liquid chromatography (HILIC), NH_2_, and hydrophilic–lipophilic balance (HLB), were supplied by Agilebio Technology Co., Ltd. (Suzhou, China). The magnetic adsorbents were based on a polystyrene backbone, possessing a specific surface area of 600–750 m^2^ g^−1^, an average pore diameter of 80–100 Å, and an ion exchange capacity of 0.5–0.8 mmol g^−1^. Acetonitrile (ACN), ammonium acetate, methanol (MeOH), formic acid, ammonium hydroxide (NH_4_OH), and sodium chloride (NaCl) were obtained from Kermel Chemical Reagent Co., Ltd. (Tianjin, China).

### 2.2. Blank Sample Preparation

Blank sugarcane samples were obtained from local markets in Shanghai, China. The sugarcane samples were peeled, cut into about 3 cm sections, homogenized, and stored at −18 °C. Sugarcane juice was prepared by mechanical pressing of the homogenized sugarcane sample and subsequently stored at −18 °C.

### 2.3. Solvent Extraction Procedure

Briefly, 2.0 g of sample was extracted with 4 mL of ACN in a 15 mL centrifuge tube via ultrasonication for 5 min. After the addition of 1 g of NaCl, the mixture was vortexed (2000 rpm, 1.5 min) and centrifuged (10,000 rpm, 5 min). Then, 2 mL of the supernatant was transferred into a new 15 mL centrifuge tube and diluted with 1 mL of distilled water. The solution was concentrated to about 0.6 mL under nitrogen at 40 °C to obtain the concentrated sample solution. Extraction yield (EY) was used to evaluate the efficiency of the solvent extraction step during optimization experiments, and was calculated as follows:

(1) $$EY=\frac{C_{1}V_{1}}{C_{0}V_{0}}\times 100\%$$

where *C*_0_ and *C*_1_ are the concentrations of 3-NPA in the spiked standard solution and in the ACN extract before MSPE, respectively. *V*_0_ and *V*_1_ are the volumes of the spiked standard solution and ACN extract, respectively.

### 2.4. MSPE Procedure

MSPE procedures were performed in 2 mL centrifuge tubes. Initially, 10 mg of MCX adsorbent was sequentially pre-activated using 1 mL of MeOH and 1 mL of 2% (*v*/*v*) formic acid aqueous solution. After magnetic separation with an external magnet positioned at the bottom of the centrifuge tube, the supernatant was removed. Subsequently, the adsorbent was added to the prepared sample concentrate, followed by vortex mixing for 1 min and another magnetic separation process. Afterward, the retained 3-NPA was eluted with 1 mL of ACN under vortex agitation for 1 min. Finally, the eluate collected after magnetic separation was filtered through a 0.22 μm membrane before HPLC-MS/MS analysis. The schematic illustration of the full sample preparation procedure was shown in Figure 1. Extraction recovery (ER) was used to evaluate the efficiency of the MSPE step during optimization experiments, and was calculated as follows:

(2) $$ER=\frac{C_{e}V_{e}}{C_{std}V_{std}}\times 100\%$$

where *C_std_* and *C_e_* represent the concentrations of 3-NPA in the original sample solution and the final 1.0 mL eluate, respectively. *V_std_* and *V_e_* denote the volumes of the extraction solution (2.0 mL) and eluate (1.0 mL), respectively.

### 2.5. HPLC-MS/MS Analysis

The determination of 3-NPA was conducted using an ACQUITY UPLC system coupled with a Xevo TQ-XS MS/MS system (Waters, Milford, MA, USA). Chromatographic separation was achieved on a BEH C18 column (100 mm × 2.1 mm, 1.7 µm) maintained at 40 °C. The mobile phase consisted of 5 mM ammonium acetate aqueous solution and MeOH, delivered at a constant flow rate of 0.3 mL min^−1^. The gradient elution conditions were set as follows: 0–3 min, 5% MeOH; 3–4 min, 40% MeOH; and 4–5 min, 5% MeOH. The injection volume was fixed at 5 μL.

Mass spectrometric detection was carried out using an electrospray ionization (ESI) source operated under negative ion mode. The optimized MS parameters were as follows: source temperature, 120 °C; capillary voltage, 0.5 kV; desolvation gas flow, 1000 L h^−1^; desolvation gas temperature, 450 °C; cone voltage, 20 V; and collision gas (argon) flow rate, 0.15 mL min^−1^. Quantitative analysis was conducted under multiple reaction monitoring (MRM) mode. The precursor ion was m/z 117.9, whereas the characteristic fragment ion was m/z 46.1. Only one characteristic fragment ion was acquired for 3-NPA, as its low molecular weight and simple structure result in very limited fragmentation under collision-induced dissociation conditions, consistent with previous reports [11,13,14].

### 2.6. Method Validation

Method validation was carried out using spiked sugarcane and sugarcane juice samples. Key analytical parameters, including linearity, limit of detection (LOD), limit of quantification (LOQ), accuracy, and precision, were systematically examined. For evaluation of linearity, blank sugarcane and sugarcane juice samples were fortified with 3-NPA at seven concentration levels (0.1, 0.5, 1, 2, 10, 20, and 50 μg kg^−1^), processed according to the procedures described in Section 2.3 and Section 2.4, and then analyzed using HPLC-MS/MS. LOD and LOQ were defined as the concentrations corresponding to signal-to-noise ratios (S/N) of 3 and 10, respectively. Accuracy was assessed by calculating the relative recovery (RR) from blank samples spiked at 1, 10, and 50 μg kg^−1^. Precision was evaluated based on the relative standard deviation (RSD) values of RR obtained from intra-day and inter-day repeated analyses. RR was used to evaluate the overall extraction recovery of the complete analytical method, and was calculated using Equation (3):

(3) $$RR=\frac{C}{C_{add}}\times 100\%$$

where *C* is the concentration of 3-NPA in the spiked sample determined by HPLC-MS/MS after the entire pretreatment workflow. *C_add_* is the theoretical spiked concentration of 3-NPA in the blank matrix sample.

Matrix effects were evaluated according to the post-extraction spike approach by comparing the average peak areas of analytes in post-extraction spiked blank matrix extract (*S*_2_) with those in neat standard solution (*S*_1_) at the same concentration. The absolute matrix effect (ME%) was calculated using Equation (4).

(4) $$ME=\frac{S_{2}}{S_{1}}\times 100\%$$

A value of 100% indicates that there is no absolute ME. If the value is above 100%, there is a signal enhancement and there is signal suppression if the value is <100%.

### 2.7. Statistical Data Analysis

All data analyses were performed using GraphPad Prism 9.0.0 for Windows, GraphPad Software, San Diego, California, USA. One-way analysis of variance (one-way ANOVA) followed by Tukey’s multiple comparisons test was applied to evaluate inter-group differences. Significant differences (*p* < 0.05) were indicated by different lowercase letters, where the letter “a” denotes the highest value, with subsequent letters indicating progressively lower values. Data are presented as mean ± standard deviation (SD), with *n* = 3 for each group.

## 3. Results and Discussion

### 3.1. Optimization of Solvent Extraction Procedure

The extractant volume plays an important role in determining whether 3-NPA can be effectively extracted from sugarcane juice. Therefore, the influence of ACN volume on EY was investigated. As illustrated in Figure 2a, the EYs of 3-NPA gradually increased as the ACN volume increased from 2 mL to 4 mL, because a suitable amount of ACN provided enough extraction capacity to promote the transfer of 3-NPA into the ACN phase. However, when the ACN volume was further increased to 8 mL, the EYs decreased. This reduction was likely related to the increased solubility of matrix components in sugarcane juice caused by excessive ACN, resulting in impurity co-extraction and stronger matrix suppression [12]. Therefore, 4 mL of ACN was selected as the optimal extraction solvent volume for the following experiments.

Vortex and ultrasonic agitation are commonly applied techniques for improving extractant dispersion and accelerating the mass transfer of target compounds from the sample matrix into the organic phase [19,20,21]. In this work, the effects of vortex and ultrasonic agitation on the EYs of 3-NPA were investigated. Under both 2 mL and 4 mL ACN extraction systems, higher EYs of 3-NPA were achieved using ultrasonic agitation compared with vortex agitation (Figure 2b). The superior performance of ultrasonic treatment was mainly related to the cavitation effect, which reduced the interfacial tension between ACN and the aqueous phase and accelerated the migration of 3-NPA into the ACN phase [22]. The maximum EY was achieved when ultrasonic agitation was combined with the 4 mL extraction system.

Adequate ultrasonic duration was necessary to ensure efficient dispersion. Ultrasonic times of 5, 10, 15, 20, and 25 min were examined, and the obtained results are presented in Figure 2c. No obvious differences in the EYs of 3-NPA were observed within the tested ultrasonic time range, indicating that ultrasonic cavitation rapidly eliminated interfacial mass-transfer resistance and enabled phase partition equilibrium of 3-NPA within 5 min. The slight decline in EY observed at longer ultrasonic times may have resulted from matrix suppression caused by co-extraction of matrix impurities [22]. Thus, 5 min was selected for the subsequent experiments.

The high moisture and soluble sugar content in sugarcane juice impairs phase separation between ACN and the aqueous phase, leading to both reduced EY of 3-NPA and increased co-extraction of matrix impurities. Addition of NaCl facilitates ACN-aqueous phase separation through the salting-out effect and also adjusts the ionic strength of the extraction system to improve target partitioning, thus increasing extraction efficiency [23,24,25]. The effect of NaCl on EY was investigated by adding different amounts of NaCl into the extraction system. As shown in Figure 2d, the EY of 3-NPA increased from 63.6% to 75.1% as the NaCl amount increased from 0.5 g to 1 g. The enhancement in EY was attributed to the increased ionic strength of the aqueous phase, which facilitated the transfer of 3-NPA into the ACN phase. When the NaCl amount was further increased, the EY showed little variation because the partition equilibrium of 3-NPA between the two phases had already been achieved. Therefore, 1 g of NaCl was selected for subsequent experiments.

### 3.2. Optimization of MSPE Procedure

In the MSPE procedure, the functional groups and physicochemical properties of the magnetic adsorbent are key factors determining the extraction efficiency. Thus, the effects of adsorbent type on the extraction performance of 3-NPA were investigated in aqueous solution. Figure 3a,b show the adsorption efficiencies and corresponding elution recoveries of 3-NPA obtained using six different magnetic adsorbents (MAX, MCX, PSA, HILIC, NH_2_, and HLB), respectively. The highest adsorption efficiency was achieved when MAX was used as the adsorbent (99.9%), which could adsorb 3-NPA through strong electrostatic interactions. However, the excessively strong binding affinity between MAX and 3-NPA hindered efficient elution of the target analyte, resulting in an extremely low ER of less than 1%. The adsorption efficiencies of PSA, NH_2_, HILIC, HLB, and MCX for 3- NPA were 49.4%, 18.7%, 4.2%, 4.9%, and 45.7%, respectively, and the corresponding ERs after elution were 62.1%, 6.1%, 13.0%, 5.5%, and 70.8%, respectively.

The extraction mechanism was analyzed based on the chemical structure and physicochemical properties of 3-NPA, as well as the structural characteristics of the adsorbents. 3-NPA contains one carboxyl group, one nitro group, and a short alkyl chain (Figure S1). Its octanol-water partition coefficient XlogP is −0.5, indicating a moderately hydrophilic nature with both polar functional groups and a nonpolar alkyl segment. The surface group of MCX was shown in Figure S2. MCX was activated using 2% (*v*/*v*) aqueous formic acid, and the pH of the adsorption system was 2.4. At this pH, the carboxyl group of 3-NPA was predominantly protonated to form a neutral molecule, such that no strong electrostatic attraction occurred with the sulfonate groups on the MCX surface. The acidic proton of the carboxylic acid group can act as a hydrogen-bond donor and interact with the negatively charged sulfonate groups on the MCX adsorbent via hydrogen bonding [26,27]. Considering the hydrophobic nature of the polystyrene backbone of MCX, the aliphatic methylene alkyl chain (-CH_2_-CH_2_-) of 3-NPA can also form hydrophobic interactions with this nonpolar backbone.

To investigate the relative contributions of these two mechanisms, the adsorption efficiency of MCX and HLB was compared. They share an identical polystyrene backbone, but HLB lacks surface sulfonic acid functionalization. HLB exhibited an adsorption efficiency of only 4.9% for 3-NPA, reflecting the hydrophobic contribution from the polystyrene backbone. By contrast, MCX achieved a markedly higher adsorption efficiency of 45.7%. This enhancement was primarily attributed to the additional hydrogen-bonding interactions between 3-NPA and the surface sulfonate groups, confirming hydrogen bonding as the dominant extraction role with hydrophobic interaction serving as a secondary contributor. For PSA, the adsorption experiment was performed under neutral conditions. At this pH, 3-NPA was mostly deprotonated into anionic form; hence, anion-exchange interaction played a major role in its adsorption. Consequently, the moderate binding forces between MCX or PSA and 3-NPA enabled efficient elution and satisfactory ER. Therefore, the extraction performances of MCX and PSA magnetic extraction materials for 3-NPA in sugarcane juice were further investigated.

Furthermore, the extraction efficiencies of MCX and PSA toward 3-NPA in sugarcane juice were assessed. As presented in Figure 4a, unlike the ERs of MCX and PSA obtained in aqueous solution (70.8% and 62.1%, respectively), the ER of MCX for 3-NPA in the sugarcane matrix reached 71.6%, which was nearly 8 times higher than that of PSA (9.0%). These findings suggested that the MCX adsorbent exhibited excellent anti-matrix interference capability. A similar performance of MCX has also been described in earlier studies on drug analysis in complex biological matrices [28,29,30]. The weak extraction performance of PSA was mainly attributed to abundant polar organic acids and carbohydrate impurities in sugarcane, which strongly competed with 3-NPA for the limited active adsorption sites on PSA, thereby reducing its extraction ability for 3-NPA [31]. By comparison, the negatively charged sulfonic acid groups on the MCX surface can exert electrostatic repulsion against the negatively charged fractions of matrix impurities, such as dissociated organic acids and acidic oligosaccharides, which is inferred to contribute to the reduced competitive adsorption [30]. Therefore, MCX was chosen as the adsorbent for extraction of 3-NPA from sugarcane juice.

The dosage of adsorbent was another important factor affecting the ER of 3-NPA [32]. As shown in Figure 4b, when the amount of PSA was raised from 5 mg to 25 mg, the ERs of 3-NPA remained low and nearly unchanged (8.7–9.9%), suggesting that serious matrix interference largely weakened the extraction ability of PSA. In the case of MCX, the ER of 3-NPA markedly increased from 52.9% to 72.1% as the amount of MCX rose from 5 mg to 10 mg, and then became relatively stable when the amount further increased to 25 mg (Figure 4c). Hence, 10 mg MCX was selected for the following experiments.

The elution solvent strongly influences the interaction between the adsorbent and 3-NPA, and therefore determines the ER of MSPE. The elution abilities of ACN, MeOH, 0.5% NH_4_OH-ACN, 1% NH_4_OH-ACN, 0.5% NH_4_OH-MeOH, and 1% NH_4_OH-MeOH toward3-NPA were examined, and the obtained results are displayed in Figure 4d. The highest ER (97.6%) was achieved when ACN was employed as the eluent, whereas MeOH provided an ER of 87.6%. Both ACN and MeOH were capable of efficiently desorbing the target analyte from the adsorbent through disruption of hydrophobic interactions and hydrogen bonding, which agrees well with the extraction mechanism proposed earlier [17]. ACN (logP = −0.34) possesses stronger hydrophobicity than MeOH (logP = −0.76), leading to better elution performance. After addition of 0.5% and 1% NH_4_OH to ACN and MeOH, the ERs of 3-NPA obviously decreased. This reduction was likely due to two reasons. First, the highly polar NH_4_OH lowered the hydrophobicity of the elution system and weakened the capability of the organic solvents to disrupt hydrophobic interactions. Second, 3-NPA could undergo degradation under alkaline conditions, which additionally decreased the ER [13]. The stability of 3-NPA under alkaline conditions was evaluated. Ammonia solution was added to a 3-NPA standard solution to simulate the alkaline extraction environment, and changes in chromatographic response were monitored by LC-MS/MS in MRM mode over time. As shown in Figure S3, a new split peak appeared at approximately 0.77 min immediately after ammonia addition. With increasing incubation time, the peak intensity of intact 3-NPA decreased gradually, while the peak at 0.77 min increased correspondingly. After approximately 43 min of exposure to alkaline conditions, the parent peak of 3-NPA almost vanished, indicating nearly complete degradation. This experimental result confirms that 3-NPA is unstable under alkaline conditions and prone to rapid degradation. Therefore, ACN was finally selected as the elution solvent.

### 3.3. Method Validation and Matrix Effects Evaluation

The developed MCX-MSPE-HPLC-MS/MS method was validated under optimized conditions. Good linearity was obtained within the investigated concentration range, and the R^2^ values of 3-NPA were both 0.999 in sugarcane and sugarcane juice (Table 1). The LOD and LOQ of 3-NPA in sugarcane juice were 0.02 μg kg^−1^ and 0.1 μg kg^−1^, respectively, while those in sugarcane were 0.04 μg kg^−1^ and 0.12 μg kg^−1^, respectively. The RRs at three fortified levels ranged between 96.9% and 105.4%. The intra-day and inter-day RSDs were within 1.4–9.7% and 1.1–6.6%, respectively. Furthermore, the accuracy and precision of the developed method at the LOQ level were evaluated. Intra-day and inter-day assays showed RR ranging from 85.8% to 106.3%, with RSDs of 10.5% to 17.8%, which were consistent with the general characteristics of trace detection at near-limit concentrations (SANTE/11312/2021). These findings demonstrated that the established MCX-MSPE-HPLC-MS/MS method possessed satisfactory accuracy and precision and could be effectively applied for determination of 3-NPA in sugarcane and sugarcane juice.

The absolute matrix effect of 3-NPA in sugarcane extracts determined by UPLC-MS/MS after MCX-MSPE cleanup is shown in Figure S4. The ME of 3-NPA in the crude extract before MSPE cleanup was 39.4%, indicating severe ion suppression from co-extracted matrix components. After MSPE cleanup with MCX adsorbent, the ME improved to 57.06%. This demonstrates that the MSPE procedure effectively removed a portion of the interfering matrix constituents and alleviated the matrix suppression effect. Electrostatic repulsion from the negatively charged sulfonate groups on the adsorbent surface was proposed as a contributing mechanism underlying this observed partial cleanup. Although a moderate degree of matrix effect remained, it could be corrected for to a certain extent by matrix-matched calibration.

For 3-NPA, quantification relies on a single characteristic fragment ion (m/z 46.1), which brings an inherent limitation in analyte structural confirmation and a corresponding risk of false positive identification. Complementary strategies including strict retention time matching and MCX-MSPE matrix cleanup were implemented to mitigate this risk. No discernible co-eluting interfering peaks were observed at the retention time of 3-NPA, confirming acceptable identification reliability under the current experimental system. For suspected interferences in future applications, high-resolution mass spectrometry (HRMS) can be used for further structural verification.

### 3.4. Comparison to Other Methods

The developed method was compared with previously reported analytical methods for 3-NPA determination in agricultural matrices (Table 2). Notably, the proposed method consumed only 6 mL of organic solvent, the lowest amount among all the compared approaches, whereas previously reported methods generally required 11–56 mL. The whole sample pre-treatment procedure was completed within approximately 50 min, less than half the time taken by most existing methods that usually need more than 100 min. In addition, the developed method exhibited markedly lower detection limits and satisfactory relative recoveries, showing improved sensitivity and reliable quantification performance compared with other HPLC-MS/MS and GC-MS/MS methods.

### 3.5. Real Sample Analysis

Seventeen sugarcane samples were obtained from local markets in Shanghai Pudong New Area, China, and analyzed using the proposed MCX-MSPE-HPLC-MS/MS method. Each sample was separated into two portions, including the 10–40 cm section above the root and the remaining middle-upper section, and both parts were analyzed independently. As listed in Table S1, 3-NPA was not detected in either section among all 17 samples. Representative MRM chromatograms (Figure S5) showed no target peak at the retention time of 3-NPA (0.84 min), and no interfering substances were observed in the corresponding time window. Given that contamination of 3-NPA in sugarcane mainly originates from *Arthrinium* spp. infection [33], representative samples were subjected to a one-week refrigerated storage observation to check for potential fungal infestation. After one week of storage, no red spots indicative of *Arthrinium* spp. infection appeared on the samples (Figure S6), and 3-NPA remained undetectable. This suggests the collected samples might be free of *Arthrinium* spp. infection, most likely because sampling was conducted during the sugarcane harvest season (December), and the samples had not undergone long-term storage that would promote fungal growth. Admittedly, given the unavailability of naturally contaminated positive samples in this study, the analytical performance of the developed method in naturally incurred matrices has not been demonstrated. Furthermore, the limited samples restrict the ability to fully reflect the overall 3-NPA contamination status of commercial sugarcane. Accordingly, to fully characterize both the practical applicability of the method in naturally contaminated samples and the 3-NPA contamination profile in sugarcane, large-scale monitoring studies across broader geographical regions are required in the future.

## 4. Conclusions

The first application of MCX-based MSPE coupled with HPLC-MS/MS was demonstrated for efficient extraction and reliable determination of 3-NPA in sugarcane and sugarcane juice. Effective extraction of 3-NPA was achieved by the mixed strong cation exchange reversed-phase MCX adsorbent through hydrogen bonding interactions and hydrophobic interactions. Meanwhile, matrix interference in sugarcane samples was partially mitigated, and electrostatic repulsion from the negatively charged sulfonate groups on the adsorbent surface was proposed as a contributing mechanism for the observed partial cleanup effect. Under optimized conditions, the developed method was successfully used for determination of 3-NPA in sugarcane samples and exhibited satisfactory linearity, precision, and accuracy. Compared with previously reported methods, the developed MCX-MSPE technique features shorter sample pretreatment time, lower organic solvent consumption, and better analytical performance. This work provides a new strategy for trace analysis of 3-NPA and offers potential technical support for food safety assessment of sugarcane and related products.

## Figures and Tables

**Figure 1:**
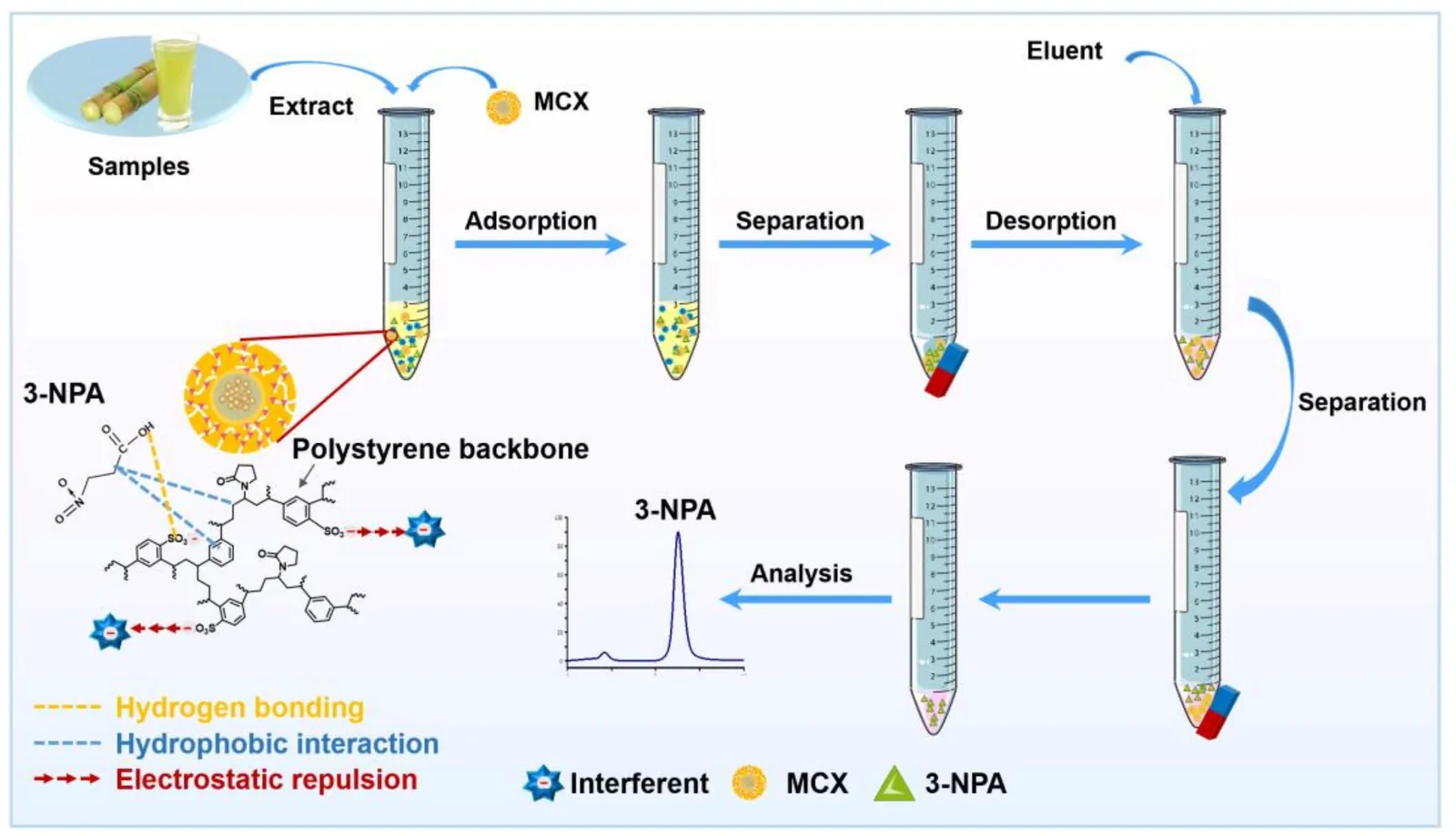
Schematic illustration of the sample preparation procedure.

**Figure 2:**
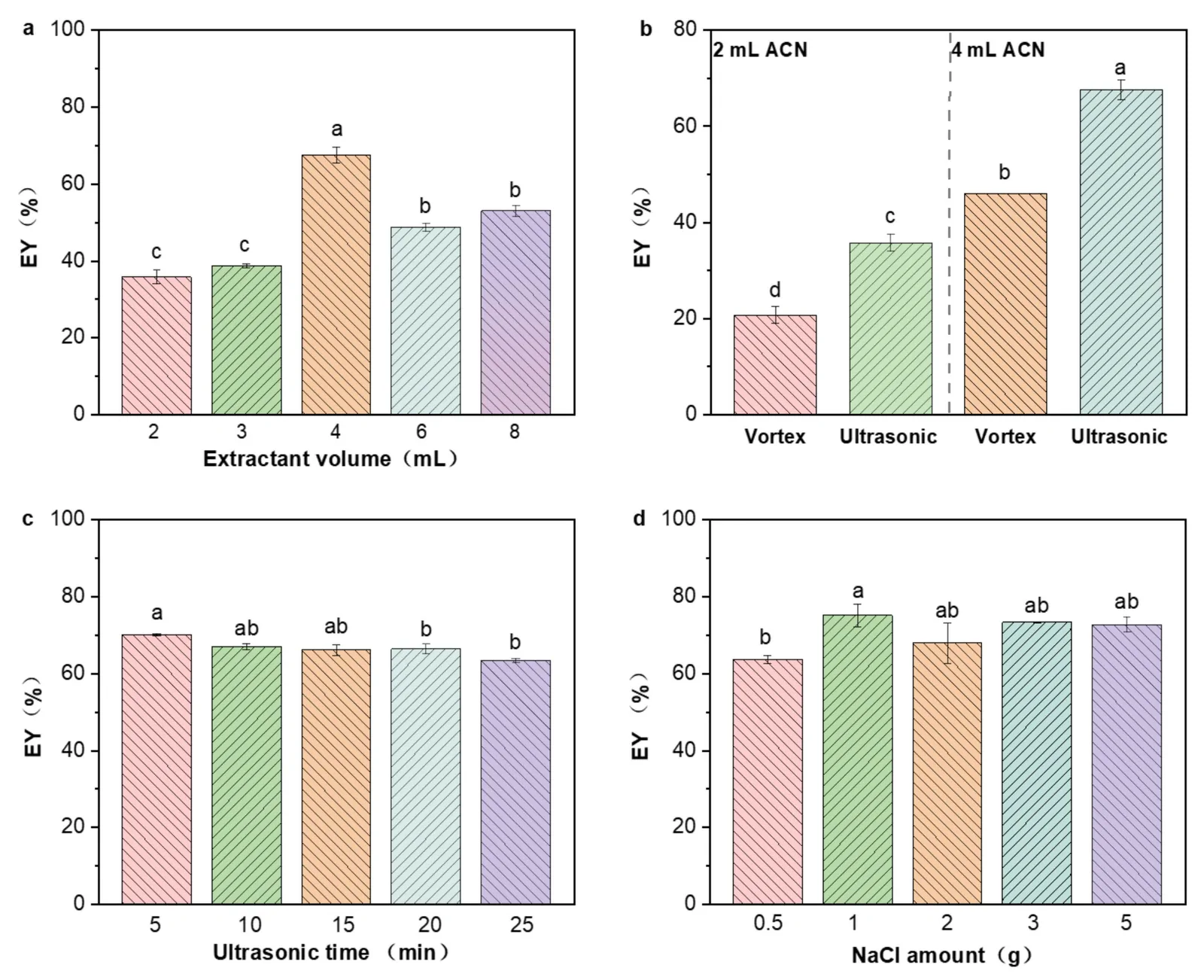
The effects of volume of extractant (**a**), type of agitation (**b**), ultrasonic time (**c**), and NaCl amount on EY. (Conditions: (**a**) ultrasonic time, 20 min; NaCl, 2g; (**b**) extractant volume, 2 mL and 4 mL; other conditions are the same as (**a**); (**c**) extractant volume, 4 mL; other conditions are the same as (**a**); (**d**) ultrasonic time, 5 min; other conditions are the same as (**c**)). Data are presented as mean ± SD. All measurements were carried out with three technical replicates (*n* = 3). One-way ANOVA was performed to evaluate inter-group differences for data shown in the above figures, followed by Tukey’s multiple comparisons test. Different lowercase letters (a–d) indicate significant differences (*p* < 0.05), whereas groups sharing the same letters show no significant difference.

**Figure 3:**
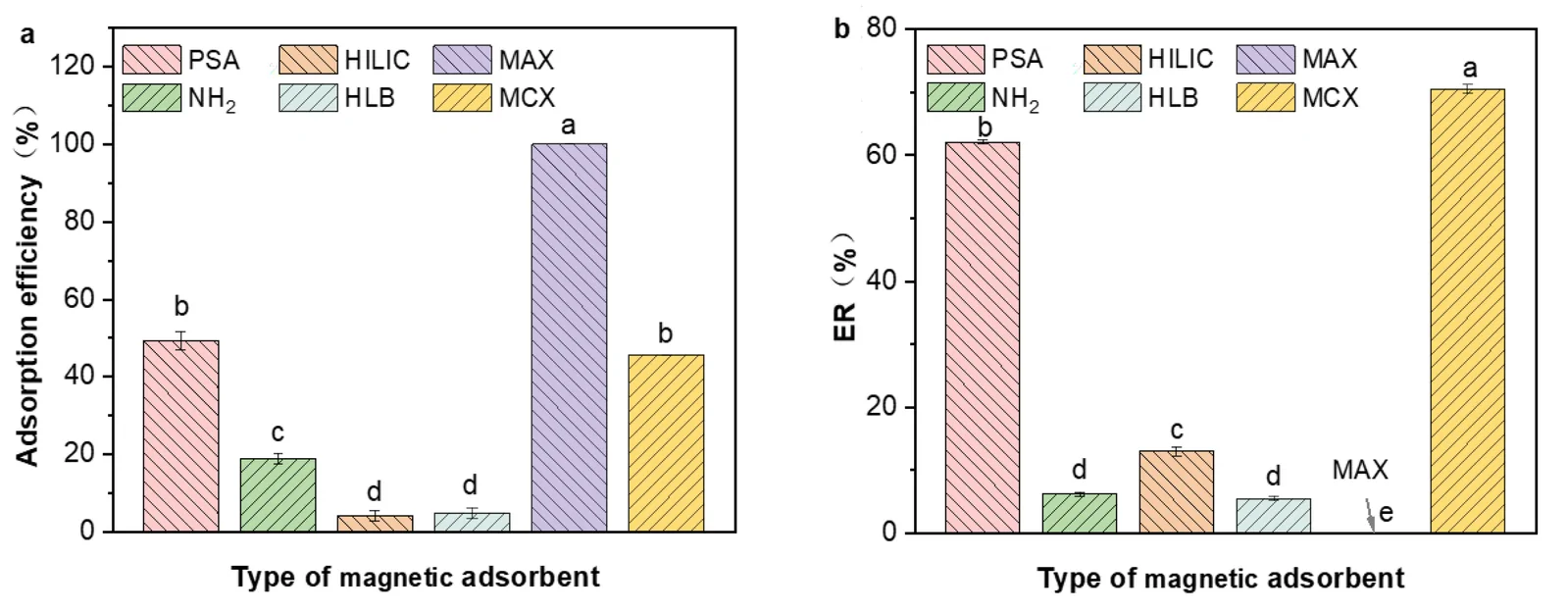
The effects of type of magnetic adsorbents on the adsorption efficiency (**a**) and extraction efficiency (**b**) of 3-NPA in aqueous solution. (Conditions: (**a**) magnetic adsorbents, 20 mg; adsorption time, 1 min; (**b**) eluent, 1% NH_4_OH-MeOH; eluent volume, 1 mL; desorption time, 1 min; other conditions are the same as (**a**)). Data are expressed as mean ± SD (*n* = 3). Inter-group differences were determined by one-way ANOVA followed by Tukey’s multiple comparisons test. Values labeled with different lowercase letters (a–e) are significantly different (*p* < 0.05), and groups bearing identical letters exhibit no statistical difference.

**Figure 4:**
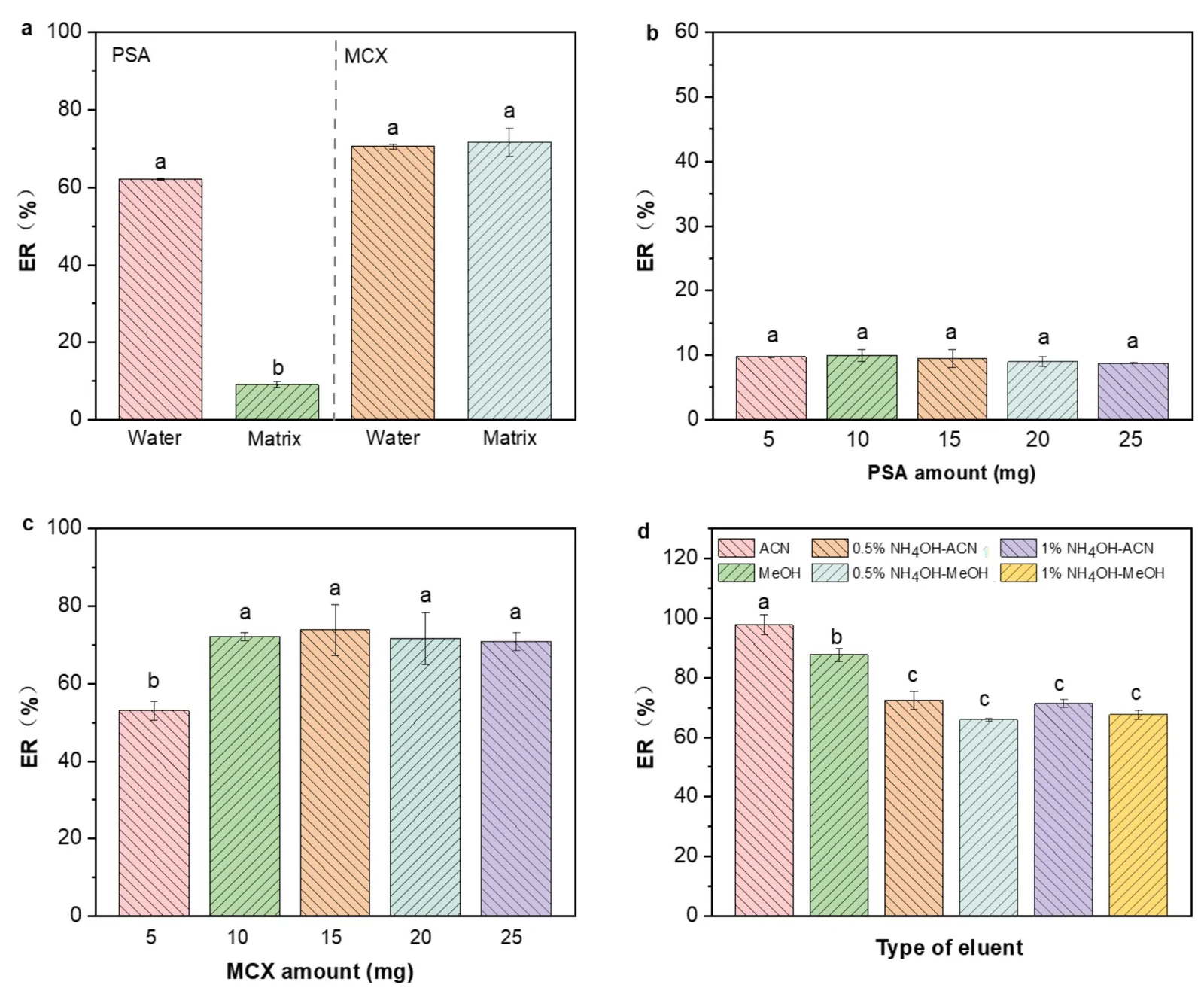
The effects of matrix (**a**), PSA amount (**b**), MCX amount (**c**) and eluent type (**d**) on ER (Conditions: (**a**) magnetic adsorbents, 20 mg; adsorption time, 1 min; eluent, 1% NH_4_OH- MeOH; eluent volume, 1 mL; desorption time, 1 min; (**b**) sugarcane juice matrix; other conditions are the same as (**a**); (**c**) sugarcane juice matrix; other conditions are the same as (**a**); (**d**) magnetic adsorbent, MCX; other conditions are the same as (**c**)). Data are presented as the mean ± SD (*n* = 3). Statistical analysis was performed as described in Figure 2. Different lowercase letters (a–c) denote significant differences at *p* < 0.05. Groups sharing the same letter indicate no significant difference between groups.

**Table 1:** Analytical performance of the proposed method.

Samples	R^2^	LOD (μg kg^−1^)	LOQ (μg kg^−1^)	Intra-Day (*n* = 6)			Inter-Day (*n* = 3)		
				RR (%) ± RSD (%)			RR (%) ± RSD (%)		
				1 μg kg^−1^	10 μg kg^−1^	50 μg kg^−1^	1 μg kg^−1^	10 μg kg^−1^	50 μg kg^−1^
Sugarcane juice	0.999	0.02	0.1	98.0 ± 9.7	102.3 ± 9.6	99.3 ± 2.7	96.9 ± 6.6	105.4 ± 2.8	97.8 ± 3.8
Sugarcane	0.999	0.04	0.12	102.4 ± 6.5	97.8 ± 5.2	99.8 ± 1.4	100.0 ± 1.1	98.6 ± 1.3	100.2 ± 2.1

**Table 2:** Comparison to other determination methods.

Sample	Organic Solvent (mL)	Time (min)	Cleanup	Analytical Method	Linear Range (μg kg^−1^)	RR (%)	LOD (μg kg^−1^)	Ref.
Sugarcane	17	>120	NH_2_-SPE	HPLC-MS/MS	10–100	80.0–96.8	3.6	[13]
Sugarcane	11 (5 mL toluene)	>170	QuEChERS	GC-APCI-MS/MS	1–100	75.1–90.2	2.0 (LOQ)	[6]
Sugarcane	56	≈137	PSA-SPE	HPLC-MS/MS	10–1000	97.5–101.2	2.7	[14]
Fabaceae plants	26.9	>55	C_18_-E-SPE	HPLC-MS/MS	6–6000	86.8–98.1	7.7	[11]
Cassava	≈16	>100	/	HPLC-MS/MS	-	80.1–80.7	1.0	[12]
Sugarcane juice	6	≈50	MCX-MSPE	HPLC-MS/MS	0.1–50	96.9–105.4	0.02	This work
Sugarcane	6	≈50	MCX-MSPE	HPLC-MS/MS	0.1–50	97.8–102.4	0.04	This work

## Data Availability

The original contributions presented in this study are included in the article/Supplementary Materials. Further inquiries can be directed to the corresponding authors.

## Supplementary Materials

The following supporting information can be downloaded at https://www.mdpi.com/article/10.3390/foods15193466/s1: Figure S1. The structure of 3-NPA. Figure S2. Chemical structure of MCX. Figure S3. MRM chromatograms of 3-NPA in neutral water (a) and in 0.1% ammonia aqueous solution at different times: 11:07 (b), 11:11 (c), 11:15 (d), 11:18 (e), and 11:50 (f). Figure S4. MRM chromatograms of 3-NPA in pre-extraction blank matrix (a), post-extraction blank matrix (b) and acetonitrile solution (c). Figure S5. MRM chromatograms of representative sugarcane samples: (a) middle-upper section; (b) 10–40 cm section above the root. Figure S6. Photos of the upper part (top) and the root part (bottom) of the representative sugarcane samples after being refrigerated at 4 °C for one week. Table S1. Contents (μg kg^−1^) of 3-NPA in 17 sugarcane samples.

## Abbreviations

The following abbreviations are used in this manuscript:

3-NPA	3-Nitropropanoic acid
ACN	Acetonitrile
ER	Extraction recovery
EY	Extraction yield
HILIC	Hydrophilic interaction liquid chromatography
HLB	Hydrophilic–lipophilic balance
LOD	Limit of detection
LOQ	Limit of quantification
MAX	Mixed strong anion exchange
MCX	Mixed strong cation exchange
MeOH	Methanol
ME	Matrix effect
MSPE	Magnetic solid-phase extraction
NH_4_OH	Ammonium hydroxide
PSA	Primary secondary amine
RR	Relative recovery
RSD	Relative standard deviation
SPE	Solid-phase extraction
