# Supplementary material for: Effective Extraction and Determination of 3-Nitropropanoic Acid in Sugarcane Samples Based on Magnetic Solid-Phase Extraction Coupled with HPLC-MS/MS
Source: Foods. 2026 Sep 28;15(19):3466. doi: 10.3390/foods15193466 (PMC13664478; doi:10.3390/foods15193466)
Supplement: Supplementary file 1 [file foods-15-03466-s001.zip › foods-4522621-supplementary.pdf]

Effective extraction and determination of 3-nitropropanoic acid in sugarcane samples based on magnetic solid-phase extraction coupled with HPLC-MS/MS

Yaqi Zhang 1,2,†, Jing Dai 1,†, Yijing Zhao 1, Aibo Wu 2, Lijun He 3 , Lei Zheng 1, Wei Min 1, Jiang Liang 4,*, Na Liu 2,* and Lipeng Hao 1,*

1 Shanghai Pudong New Area Center for Disease Control and Prevention (Shanghai Pudong New Area Health Supervision Institute), Shanghai 200136, China

2 Shanghai Institute of Nutrition and Health, University of Chinese Academy of Sciences, Chinese Academy of Sciences, Shanghai 200031, China

3 School of Chemistry and Chemical Engineering, Henan University of Technology, Zhengzhou 450001, China

4 China National Center for Food Safety Risk Assessment, Beijing 100022, China

* Correspondence: liangjiang@cfsa.net.cn (J.L.); liuna@sinh.ac.cn (N.L.); lphaomail@163.com (L.H.)

† These authors contributed equally to this work.

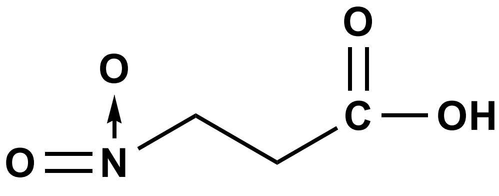

Figure S1 The structure of 3-NPA

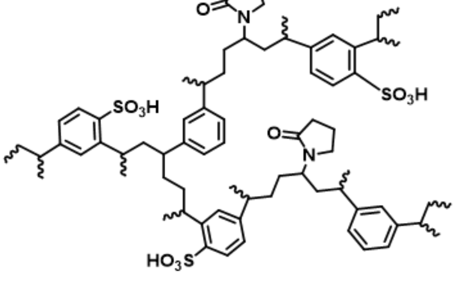

Figure S2 Chemical structure of MCX

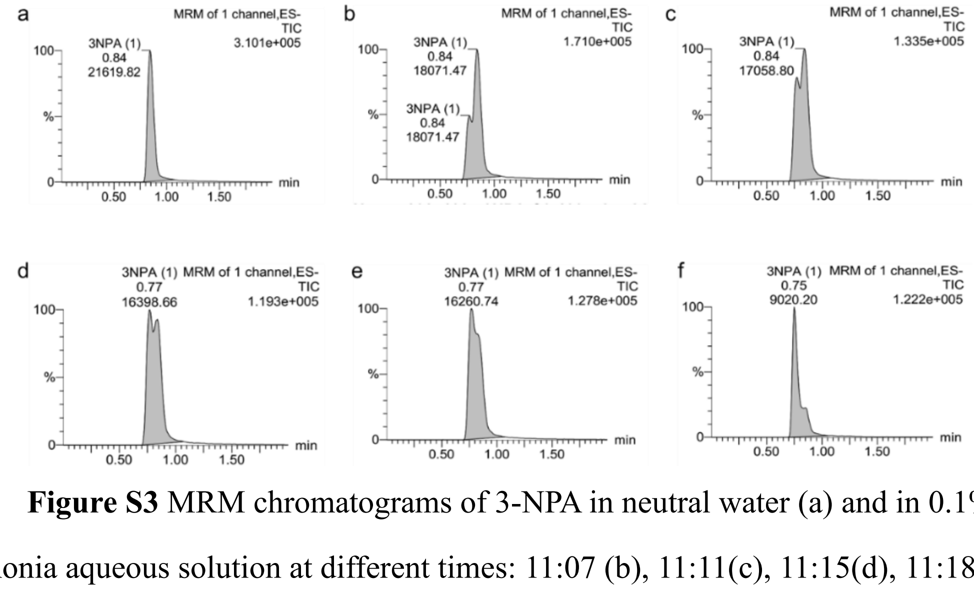

Figure S3 MRM chromatograms of 3-NPA in neutral water (a) and in 0.1% ammonia aqueous solution at different times: 11:07 (b), 11:11(c), 11:15(d), 11:18(e), and 11:50 (f). The retention of 3-NPA is 0.84 min.

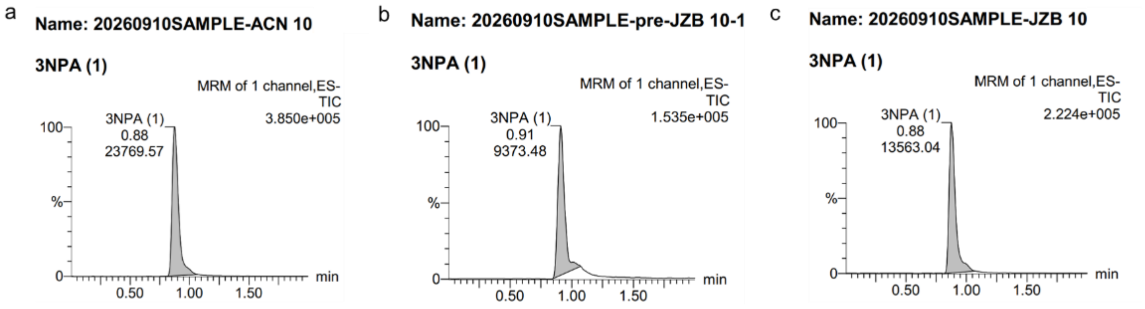

Figure S4 MRM chromatograms of 3-NPA in acetonitrile solution (a), pre-extraction blank matrix (b) and post-extraction blank matrix (c).

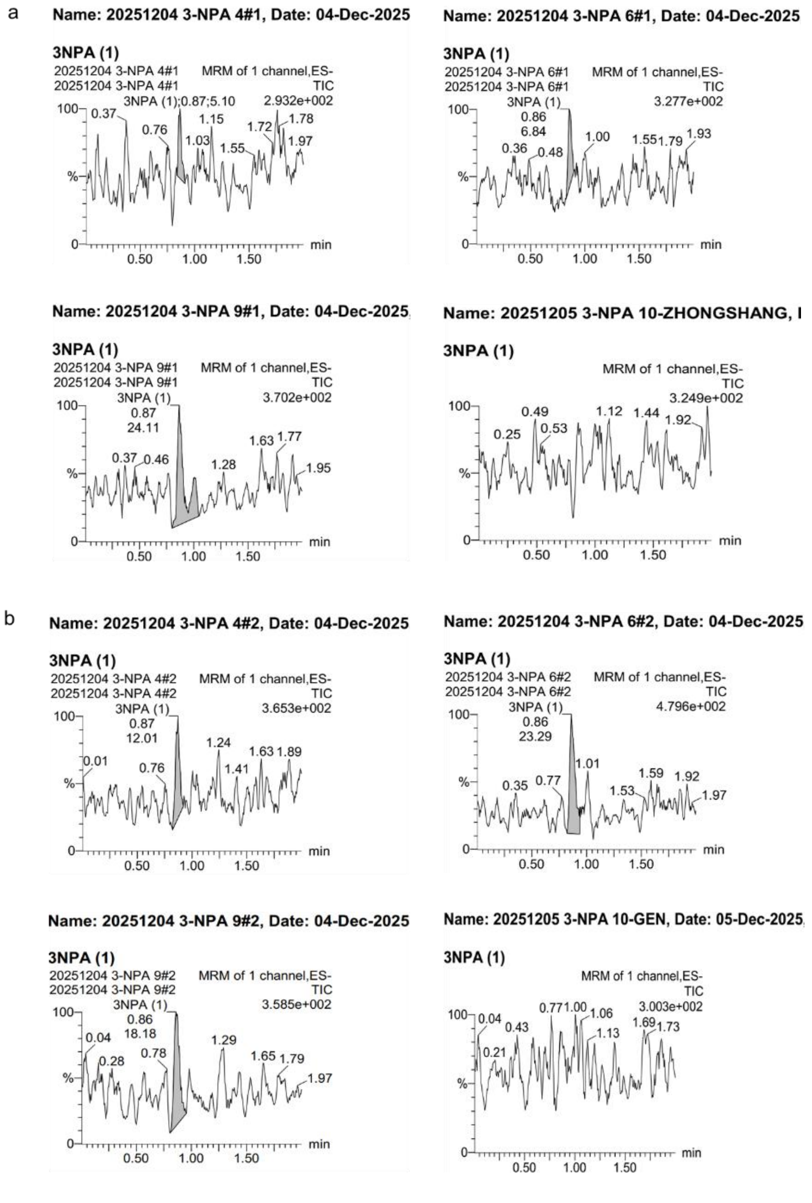

Figure S5 MRM chromatograms of representative sugarcane samples. (a) middle-upper section; (b) 10–40 cm section above the root.

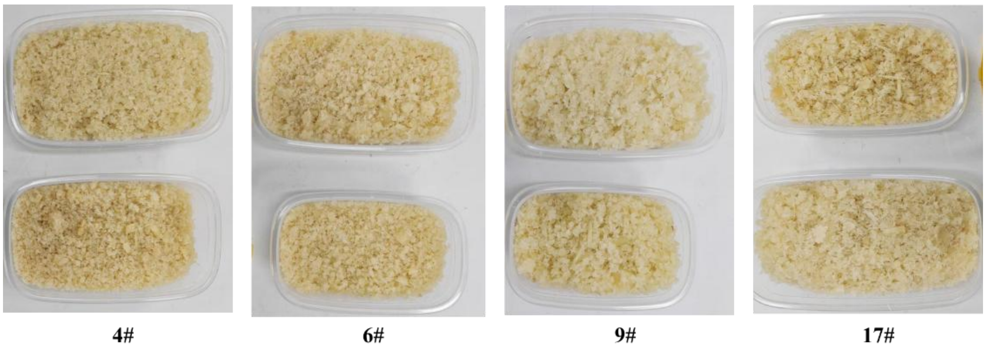

Figure S6 Photos of the upper part (top) and the root part (bottom) of the representative sugarcane samples after being refrigerated at 4 °C for one week.

Table S1 Contents ($\mu g\ kg^{-1}$) of 3-NPA in 17 sugarcane samples.

Samples	No.	A*	B*	Samples	No.	A*	B*	Samples	No.	A*	B*
Green-skinned sugarcane	1	ND*	ND	Purple-skinned sugarcane	10	ND	ND	Red-skinned sugarcane	15	ND	ND
	2	ND	ND		11	ND	ND		16	ND	ND
	3	ND	ND		12	ND	ND		17	ND	ND
	4	ND	ND		13	ND	ND				
	5	ND	ND		14	ND	ND				
	6	ND	ND								
	7	ND	ND								
	8	ND	ND								
	9	ND	ND								

*A: the 10–40 cm section above the root

*B: the remaining middle-upper section

* ND = Not Detected

* LOQ of the present method is 0.12 $\mu g\ kg^{-1}$
